# You are what you eat: a systematic review exploring the interaction between Brazilian sand flies and their vertebrate food sources

**DOI:** 10.1590/0074-02760240055

**Published:** 2024-08-30

**Authors:** Felipe Dutra-Rêgo, Michelli Santos da Silva, Ana Paula Isnard, Jansen Fernandes Medeiros, José Dilermando Andrade, Mariana Lourenço Freire

**Affiliations:** 1 Fundação Oswaldo Cruz-Fiocruz, Instituto René Rachou, Grupo de Estudos em Leishmanioses, Belo Horizonte, MG, Brasil; 2 Universidade Federal de Rondônia/Fundação Oswaldo Cruz-Fiocruz, Programa de Pós-Graduação em Biologia Experimental, Porto Velho, RO, Brasil; 3 Fundação Oswaldo Cruz-Fiocruz, Laboratório de Entomologia, Porto Velho, RO, Brasil; 4 Instituto Nacional de Epidemiologia na Amazônia Ocidental, Porto Velho, RO, Brasil; 5 Fundação Oswaldo Cruz-Fiocruz, Instituto René Rachou, Pesquisa Clínica e Políticas Públicas em Doenças Infecto-Parasitárias, Belo Horizonte, MG, Brasil

**Keywords:** sand fly, blood-feeding habits, systematic review, Brazil

## Abstract

Sand flies play a crucial role as vectors of bacteria, viruses, and protists, with *Leishmania* being the most notable among them, transmitted to vertebrate hosts during blood feeding. Understanding the feeding behaviours of sand flies is imperative for gaining insights into their eco-epidemiological roles in the transmission of these infectious agents. This systematic review aimed to answer the question *‘What are the blood-feeding sources identified in Brazilian sand flies?*’ to provide an analysis of their blood-feeding habits. The diverse range of at least 16 vertebrate orders identified as blood sources for 54 sand fly species across different geographic regions was summarised, and the factors potentially associated with the risk of bias in the included studies were analysed. The findings broaden the discussion concerning methods used to identify blood meal sources and shed light on the implications of sand fly feeding behaviours for the transmission dynamics of *Leishmania*.

Vector-borne diseases present significant public health challenges globally, necessitating a thorough investigation of the intricate interactions between arthropod vectors and vertebrate hosts.^1^ Among these vectors, sand flies (Diptera: Psychodidae) have garnered considerable attention due to their role in transmitting *Leishmania*, the causative agents of leishmaniasis, a widespread tropical disease with substantial global health implications.^2^ ^,^ ^3^ The expanding geographical range of leishmaniasis, driven by factors such as urbanisation,^4^ ^,^ ^5^ climate change,^6^ ^,^ ^7^ and human migration,^8^ ^,^ ^9^ underscores the urgent need to understand the determinants of disease transmission, as current control strategies have been inadequate in containing its spread.^10^

Despite their significance as vectors of *Leishmania*, studies on the feeding behaviours of sand flies, particularly in Brazil, a biodiversity hotspot, have only gained attention in the last decades. This region offers a unique opportunity to investigate sand fly-host interactions. Understanding the feeding habits of sand flies is paramount, as these behaviours have been shown to be influential in vector longevity,^11^ fecundity,^12^ oviposition,^13^ and vectorial capacity.^14^ Moreover, the intricate network of interactions between sand flies and vertebrates plays a crucial role in shaping the dynamics of *Leishmania* transmission.^15^ ^,^ ^16^

This systematic review aims to gather data on the analysis of the blood-feeding behaviour of Brazilian sand flies, with an emphasis on their role as putative vectors of *Leishmania*. Additionally, concerns regarding bias in the information within the included studies were evaluated using a specifically developed tool to assess this type of study.

## MATERIALS AND METHODS

*Protocol* - This review was systematically conducted following the methodological principles outlined in the Cochrane Handbook,^17^ with adaptations for this type of study,^18^ and the Preferred Reporting Items for Systematic Reviews and Meta-Analyses (PRISMA) guidelines.^19^

*Eligibility criteria* - The systematic review was guided by the following research question: *‘What are the blood feeding sources identified in Brazilian sand flies?*’ The article selection process and inclusion criteria followed the PICo framework (population, phenomenon of interest, and context): **(P)** Phlebotominae, **(I)** blood-feeding sources, and **(Co)** Brazil. Original research articles reporting the identification of blood sources in wild-caught female sand flies from Brazil were included. Articles describing techniques for identifying the blood sources of sand flies without field collections, outlining the attractiveness of sand flies to various baits (rodent, lizard, chicken, etc.), and/or reporting the identification of sand fly food sources outside Brazil were excluded.

*Search strategy* - Structured searches were conducted in three databases: MEDLINE (PubMed), Web of Science, and the Virtual Health Library (VHL). In each database, keywords associated with “Phlebotominae”, “Blood feeding”, and “Brazil” were combined using Boolean operators (AND, OR). Details about the search strategy employed in each database are available in the Supplementary data (Table). Articles published up to October 10, 2023, were included without any restrictions on the publication date. Furthermore, supplementary searches of the reference lists of the included articles were undertaken to ensure a thorough exploration of the literature.

Data retrieved from each database underwent initial processing in Mendeley Reference Management to identify and eliminate duplicate files (the same study found in different databases). Following this, the records were transferred to Rayyan for screening based on titles and abstracts.^20^ Two independent reviewers (MSS, API) conducted the screening process, following the predefined inclusion and exclusion criteria. Discrepancies were resolved through consensus, or by an additional reviewer (FDR) if an agreement was not reached. The full texts of selected studies were thoroughly examined to validate their eligibility, extract relevant data, and ensure that exclusion criteria were not applicable.

*Data extraction* - The primary characteristics of the studies, including details about the population, phenomenon of interest, and context, were independently extracted by three reviewers (FDR, MSS, API) and subsequently cross-referenced to confirm all obtained data. Extracted data included sand fly collections, the Brazilian states where sand flies were collected, the overall number of collected sand flies, the total count of collected and engorged females, the variety of vertebrate species identified as blood sources, and specific details concerning the methodology employed for blood meal identification. Sand fly nomenclature and genera abbreviations used here followed Galati^21^ and Marcondes,^22^ respectively. Citations involving sand fly species complexes, specifically those morphologically indistinguishable, and synonyms were presented in accordance with the original records.

*Risk of bias* - To enable a critical and transparent analysis of the results obtained, an effort was made to examine potential sources of bias in the included articles related to three main domains: (I) Sand fly identification; (II) Sample quality; and (III) Methods for food source identification. For this analysis, signalling questions related to each domain were proposed (Table I) and analysed in all included articles.

**TABLE I:** Domains and signaling questions used to analyze the risk of bias in the included articles

Domain	Sand fly identification and processing	Sample quality	Methods for food source identification
Signaling questions (Yes/No/Unclear)	Was any taxonomic key used? Was the processing of sand flies carried out adequately?	Was the characterisation of blood feeding in females conducted properly? Was the dissection and the preservation of females carried out adequately?	Was the methodology used to identify the food source appropriate? Were appropriate controls used? Were cut off points predefined?
Description	The classification key used to identify sand flies was informed and how the insects were dissected, and stored to preserve it until processing was described	The feeding level of the females was evaluated, the dissection and preservation methods were presented	The methodology used was adequately described, controls were used, and definitions of positive results were presented
Risk of bias (High/Low/Unclear)	Could the identification and processing of sand flies have introduced bias?	Could the verification of blood feeding or its interpretation have introduced bias?	Could the conduct or interpretation of the food source have introduced bias?

## RESULTS

**Fig. 1:**
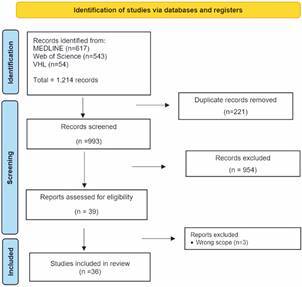
flow diagram illustrating the study selection process according to Preferred Reporting Items for Systematic Reviews and Meta-Analyses (PRISMA) guidelines.

*Literature search* - A total of 1,214 articles were initially identified from the databases, with 221 excluded due to duplication. After analysing titles and abstracts, 39 articles were selected for full-text reading. Among these, 36 articles were included, and a subsequent rigorous examination of reference lists did not yield the inclusion of any additional articles. Flow diagrams outlining each step of this systematic review, following the PRISMA guidelines, are presented in Fig. 1.

*Descriptive analysis of included studies* - The characteristics of all included studies are presented in Table II, including information about the blood sources of sand flies from various Brazilian states. Light traps were utilised in most of the included studies (33/36), while manual collections (2/36) or Shannon traps (1/36) were less frequently employed. The total number of collected sand flies varied between 80 and 15,457, with the percentage of engorged females among the total collected females ranging from 0.4% to 100%. The results showed a slight tendency for engorged females to be captured using manual collection instead of Shannon or light traps.

**TABLE II:** Characteristics of the studies included in the systematic review

Reference	Brazilian state	Collection	Total of collected sand flies	Total of females	Total of engorged females	Percentage of engorged females	Total of species engorged	Total of vertebrate species identified
^113^	RJ	manual and light trap	NA	370	189	51,1	1	4
^35^	BA, CE, PI	light trap	NA	609	609	100,0	1	9
^50^	AC	light trap	4473	2297	96	4,2	20	13
^51^	AC	light trap and Shannon	2517	206	33	16,0	11	1
^114^	MG	light trap	1408	525	38	7,2	1	7
^31^	PR	light trap	3357	864	862	99,8	1	8
^25^	PR	light trap and Shannon	2851	1263	93	7,4	1	3
^36^	MT	light trap	2011	657	61	9,3	1	3
^56^	MG	light trap	NA	362	192	53,0	8	4
^115^	AM	manual	NA	2569	2569	100,0	2	19
^81^	RO	light trap	1706	1227	160	13,0	9	7
^24^	MG	manual	198	107	79	73,8	2	2
^116^	MS	light trap	1542	502	24	4,8	2	3
^88^	MA	light trap	NA	70	70	100,0	1	6
^117^	MA	light trap	9853	5061	297	5,9	6	6
^27^	MA	light trap	NA	982	573	58,4	7	8
^54^	RO	light trap	15457	8788	86	1,0	4	4
^79^	RN	light trap	1768	230	81	35,2	1	2
^118^	MS	light trap	NA	83	83	100,0	2	1
^119^	SP	Shannon	NA	NA	737	-	2	6
^89^	MT	light trap	NA	2376	104	4,4	1	7
^37^	AM	manual and light trap	NA	199	199	100,0	4	5
^38^	MS	light trap	120	120	1	0,8	1	1
^120^	MS	light trap	3219	3219	355	11,0	10	4
^82^	MA	light trap	NA	NA	274	-	3	7
^121^	MG	light trap	6034	NA	108	-	3	4
^53^	RO	light trap	9535	4089	15	0,4	6	3
^48^	PA	light trap	NA	81	59	72,8	1	6
^26^	MG	light trap	161	126	38	30,2	1	4
^49^	RR	light trap	1209	704	34	4,8	4	4
^39^	PI	light trap	2089	392	58	14,8	1	2
^52^	RO	light trap	1943	887	50	5,6	3	4
^122^	RJ	light trap	80	80	76	95,0	1	3
^123^	MG	light trap	2614	524	97	18,5	2	4
^78^	RO	light trap	1331	709	7	1,0	2	3
^124^	AP	light trap, manual, and Shannon	9119	5073	138	2,7	5	6

NA: data not available.

The food source data were recovered for 13 out of the 19 genera of Brazilian sand flies, encompassing a total of at least 54 species and subspecies out of about 280 species currently described in Brazil,^21^ corresponding to approximately 19% of the total. The following genera had information available: *Bichromomyia*, *Evandromyia*, *Lutzomyia*, *Micropygomyia*, *Migonemyia*, *Nyssomyia*, *Pintomyia*, *Pressatia*, *Psathyromyia*, *Psychodopygus*, *Sciopemyia*, *Trichophoromyia*, and *Trichopygomyia*. There is a lack of information regarding the blood food source for the remaining genera, including *Brumptomyia*, *Deanemyia*, *Edentomyia*, *Expapillata*, *Martinsmyia*, and *Viannamyia*. It is noteworthy that *De. maruaga*, a troglobite species, exhibits autogenic and parthenogenic behaviour,^23^ but it is unclear whether this behaviour extends to all species within the genus.

Most of the studies (58%) demonstrated a predominant orientation towards the use of DNA-based methods, in which information regarding at least 42 species was retrieved (Table III). Among the 21 studies utilising molecular markers, 20 (95%) exclusively employed mitochondrial targets. Notably, *cytochrome b* (*cytb*) was the preferred molecular marker in 94% of the cases. The remaining two studies (6%) utilised cytochrome oxidase I (COI),^24^ and the nuclear gene prepronociceptin (PNOC) as molecular targets.^25^ For host identification, 16 articles (76%) employed Sanger sequencing, followed by polymerase chain reaction-restriction fragment length polymorphism (PCR-RFLP) (four articles, 19%), and specific primers for each host (one article, 5%).

**TABLE III:** Blood source identification of Brazilian sand fly species by polymerase chain reaction (PCR)

Sand fly	Number/Range of specimens collected	Vertebrate host	Vertebrate host order	Number/Range of sand flies feeding on the host	Molecular target	Identification of blood meal	Reference
*Bi. flaviscutellata*	86	*Canis lupus familiaris*	Carnivora	5	*cytb*	PCR-RFLP	^117^
	86	*Equus ferus caballus*	Perissodactyla	1	*cytb*	PCR-RFLP	^117^
	86	*Gallus gallus*	Galliformes	5	*cytb*	PCR-RFLP	^117^
	86-222	*Homo sapiens*	Primates	1-2	*cytb*	PCR-RFLP and Sanger sequencing	^52^ ^,^ ^53^ ^,^ ^117^
	222	*Proechimys gardneri*	Rodentia	1	*cytb*	Sanger sequencing	^52^
	86	*Sus scrofa*	Artiodactyla	9	*cytb*	PCR-RFLP	^117^
*Br. olmeca nociva*	9	*Bos taurus*	Artiodactyla	1	*cytb*	Sanger sequencing	^50^
*Ev. Cortelezzii* complex	4	*Homo sapiens*	Primates	1	*cytb*	Sanger sequencing	^56^
*Ev. evandroi*	3-18	*Canis lupus familiaris*	Carnivora	2-5	*cytb*	PCR-RFLP	^27^ ^,^ ^117^
	542	*Equus ferus caballus*	Perissodactyla	1	*cytb*	PCR-RFLP	^117^
	542	*Gallus gallus*	Galliformes	6	*cytb*	PCR-RFLP	^117^
	3	*Homo sapiens*	Primates	1	*cytb*	PCR-RFLP	^27^
	542	*Sus scrofa*	Artiodactyla	5	*cytb*	PCR-RFLP	^117^
	3-542	*Canis lupus familiaris*	Carnivora	2-5	*cytb*	PCR-RFLP	^27^ ^,^ ^117^
*Ev. lenti*	1152	*Gallus gallus*	Galliformes	2	*cytb*	PCR-RFLP	^117^
	1152	*Sus scrofa*	Artiodactyla	6	*cytb*	PCR-RFLP	^117^
	4	Rodent (generic)	Rodentia	1	*cytb*	PCR-RFLP	^27^
*Ev. saulensis*	187	*Homo sapiens*	Galliformes	8	*cytb*	Sanger sequencing	^51^
	70	*Canis lupus familiaris*	Primates	2	*cytb*	Sanger sequencing	^50^
	70	*Sus scrofa*	Artiodactyla	1	*cytb*	Sanger sequencing	^50^
*Evandromyia* sp.	1	*Dasypus novemcinctus*	Cingulata	1	*cytb*	Sanger sequencing	^49^
	30	*Tamandua tetradactyla*	Pilosa	1	*cytb*	Sanger sequencing	^50^
*Ev. termitophila*	5	*Canis lupus familiaris*	Carnivora	4	*cytb*	PCR-RFLP	^27^
*Ev. walkeri*	31	*Gallus gallus*	Galliformes	2	*cytb*	Sanger sequencing	^51^
	88	*Sus scrofa*	Artiodactyla	1	*cytb*	Sanger sequencing	^81^
*Lu. cruzi*	1454	*Homo sapiens*	Primates	22	*cytb*	Sanger sequencing	^116^
	1454	*Dasyprocta azarae*	Rodentia	1	*cytb*	Sanger sequencing	^116^
*Lu. forattinii*	57	*Canis lupus familiaris*	Carnivora	1	*cytb*	Sanger sequencing	^116^
*Lu. longipalpis*	80-938	*Bos taurus*	Artiodactyla	1	*cytb*	PCR-RFLP	^27^ ^,^ ^122^
	91-3943	*Canis lupus familiaris*	Carnivora	1-151	*cytb*	PCR-RFLP, Sanger sequencing and specific primers	^27^ ^,^ ^39^ ^,^ ^56^ ^,^ ^117^ ^,^ ^121^
	80-2522	*Equus ferus caballus*	Perissodactyla	2-11	*cytb*	PCR-RFLP	^27^ ^,^ ^117^ ^,^ ^122^
	1324	*Euphractus sexcinctus*	Cingulata	69	*cytb*	Specific primers	^79^
	91-2522	*Gallus gallus*	Galliformes	6-184	*cytb*	PCR-RFLP, Sanger sequencing and specific primers	^27^ ^,^ ^39^ ^,^ ^56^ ^,^ ^117^
	80-3943	*Homo sapiens*	Primates	1-38	*cytb*	PCR-RFLP, Sanger sequencing and specific primers	^27^ ^,^ ^38^ ^,^ ^56^ ^,^ ^79^ ^,^ ^121^ ^,^ ^122^
	938	Opossum (generic)	Didelphimorphia	4	*cytb*	PCR-RFLP	^27^
	3943	*Planigale maculata*	Dasyuromorphia	1	*cytb*	Sanger sequencing	^121^
	938	Rodent (generic)	Rodentia	47	*cytb*	PCR-RFLP	^27^
	938-2522	*Sus scrofa*	Artiodactyla	12-19	*cytb*	PCR-RFLP	^27^ ^,^ ^117^
	3943	*Turdus poliocephalus*	Passeriformes	1	*cytb*	Sanger sequencing	^121^
*Lu. sherlocki*	101	*Homo sapiens*	Primates	1	*cytb*	Sanger sequencing	^50^
	10-101	*Sus scrofa*	Artiodactyla	1-2	*cytb*	Sanger sequencing	^50^ ^,^ ^81^
*Mi. trinidadensis*	6	*Canis lupus familiaris*	Carnivora	3	*cytb*	PCR-RFLP	^27^
	6	*Homo sapiens*	Primates	1	*cytb*	PCR-RFLP	^27^
*Mg. migonei*	53	*Canis lupus familiaris*	Carnivora	15	*cytb*	Sanger sequencing	^56^
	3-53	*Gallus gallus*	Galliformes	1-28	*cytb*	Sanger sequencing	^51^ ^,^ ^56^
	53	*Homo sapiens*	Primates	6	*cytb*	Sanger sequencing	^56^
	53	*Rattus rattus*	Rodentia	2	*cytb*	Sanger sequencing	^56^
*Ny. antunesi*	81	*Choloepus didactylus*	Pilosa	4	*cytb*	Sanger sequencing	^48^
	241	*Choloepus hoffmanni*	Pilosa	1	*cytb*	Sanger sequencing	^52^
	81	*Cuniculus paca*	Rodentia	1	*cytb*	Sanger sequencing	^48^
	81	*Dasyprocta leporina*	Rodentia	1	*cytb*	Sanger sequencing	^48^
	58	*Gallus gallus*	Galliformes	3	*cytb*	Sanger sequencing	^51^
	81-1397	*Homo sapiens*	Primates	1-2	*cytb*	Sanger sequencing	^48^ ^,^ ^53^
	81	*Pteroglossus aracari*	Piciformes	2	*cytb*	Sanger sequencing	^48^
	81-2530	*Tamandua tetradactyla*	Pilosa	2-11	*cytb*	Sanger sequencing	^48^ ^,^ ^52^ ^,^ ^53^ ^,^ ^54^
*Ny. Antunesi* complex	777	*Bos taurus*	Artiodactyla	1	*cytb*	Sanger sequencing	^81^
	777	*Pecari tajacu*	Artiodactyla	1	*cytb*	Sanger sequencing	^81^
	777	*Plecturocebus bernhardi*	Primates	1	*cytb*	Sanger sequencing	^81^
	777	*Philander canus*	Didelphimorphia	3	*cytb*	Sanger sequencing	^81^
	777	*Sus scrofa*	Artiodactyla	112	*cytb*	Sanger sequencing	^81^
	777	*Tamandua tetradactyla*	Pilosa	5	*cytb*	Sanger sequencing	^81^
*Ny. intermedia*	1263	*Canis lupus familiaris*	Carnivora	10	PNOC	Sanger sequencing	^25^
	1263	*Equus ferus caballus*	Perissodactyla	1	PNOC	Sanger sequencing	^25^
	1263	*Sus scrofa*	Artiodactyla	6	PNOC	Sanger sequencing	^25^
*Ny. richardwardi*	1	*Sus scrofa*	Artiodactyla	1	*cytb*	Sanger sequencing	^81^
	7	*Tamandua tetradactyla*	Pilosa	1	*cytb*	Sanger sequencing	^52^
*Ny. shawi*	1200	*Homo sapiens*	Primates	1	*cytb*	Sanger sequencing	^50^
*Nyssomyia* sp.	265	*Homo sapiens*	Primates	1	*cytb*	Sanger sequencing	^50^
*Ny. umbratilis*	5	*Plecturocebus bernhardi*	Primates	1	*cytb*	Sanger sequencing	^81^
	5	*Sus scrofa*	Artiodactyla	2	*cytb*	Sanger sequencing	^81^
*Ny. whitmani*	24-5304	*Canis lupus familiaris*	Carnivora	8-27	*cytb*	PCR-RFLP, Sanger sequencing	^27^ ^,^ ^56^ ^,^ ^117^
	5304	*Equus ferus caballus*	Perissodactyla	12	*cytb*	PCR-RFLP	^117^
	24-5304	*Gallus gallus*	Galliformes	4-55	*cytb*	PCR-RFLP, Sanger sequencing	^27^ ^,^ ^56^ ^,^ ^117^
	24-5304	*Homo sapiens*	Primates	2-6	*cytb*	PCR-RFLP, Sanger sequencing	^27^ ^,^ ^56^ ^,^ ^117^
	25	*Philander canus*	Didelphimorphia	1	*cytb*	Sanger sequencing	^81^
	24-5304	Rodent (generic)	Rodentia	1-7	*cytb*	PCR-RFLP	^27^ ^,^ ^117^
	25-5304	*Sus scrofa*	Artiodactyla	1-33	*cytb*	PCR-RFLP, Sanger sequencing	^81^ ^,^ ^117^
*Pi. bianchigalatiae*	5	*Gallus gallus*	Galliformes	1	*cytb*	Sanger sequencing	^56^
	5	*Homo sapiens*	Primates	1	*cytb*	Sanger sequencing	^56^
*Pi. fiocruzi*	24	*Choloepus didactylus*	Pilosa	1	*cytb*	Sanger sequencing	^54^
	24	*Micrastur gilvicollis*	Falconiformes	1	*cytb*	Sanger sequencing	^54^
*Pi. fischeri*	3	*Gallus gallus*	Galliformes	1	*cytb*	Sanger sequencing	^56^
*Pi. nevesi*	75	*Gallus gallus*	Galliformes	1	*cytb*	Sanger sequencing	^51^
	16	*Homo sapiens*	Primates	1	*cytb*	Sanger sequencing	^50^
*Pi. pessoai*	4	*Gallus gallus*	Galliformes	2	*cytb*	Sanger sequencing	^56^
	495	*Homo sapiens*	Primates	1	*cytb*	Sanger sequencing	^121^
*Pi. serrana*	62	*Cuniculus paca*	Rodentia	1	*cytb*	Sanger sequencing	^50^
	62	*Coendou prehensilis*	Rodentia	1	*cytb*	Sanger sequencing	^50^
	10	*Gallus gallus*	Galliformes	1	*cytb*	Sanger sequencing	^51^
	62	*Homo sapiens*	Primates	1	*cytb*	Sanger sequencing	^50^
*Pressatia* sp.	86	*Gallus gallus*	Galliformes	2	*cytb*	Sanger sequencing	^51^
*Pa. aragaoi*	20	*Homo sapiens*	Primates	1	*cytb*	Sanger sequencing	^50^
*Pa. dendrophyla*	26	*Bos taurus*	Artiodactyla	2	*cytb*	Sanger sequencing	^53^
	3	*Sus scrofa*	Artiodactyla	1	*cytb*	Sanger sequencing	^81^
*Psathyromyia* sp.	20	*Tamandua tetradactyla*	Pilosa	1	*cytb*	Sanger sequencing	^50^
*Ps. amazonensis*	98	*Tamandua tetradactyla*	Pilosa	1	*cytb*	Sanger sequencing	^50^
*Ps. ayrozai*	509	*Dasypus novemcinctus*	Cingulata	25	*cytb*	Sanger sequencing	^49^
	2198	*Homo sapiens*	Primates	1	*cytb*	Sanger sequencing	^54^
	2198	*Tamandua tetradactyla*	Pilosa	1	*cytb*	Sanger sequencing	^54^
*Ps. carrerai*	99	*Gallus gallus*	Galliformes	1	*cytb*	Sanger sequencing	^51^
*Ps. carrerai carrerai*	456	*Bos taurus*	Artiodactyla	1	*cytb*	Sanger sequencing	^53^
	293	*Dasypus novemcinctus*	Cingulata	4	*cytb*	Sanger sequencing	^50^
	293	*Homo sapiens*	Primates	1	*cytb*	Sanger sequencing	^50^
*Ps. Chagasi* series	152	*Cuniculus paca*	Rodentia	1	*cytb*	Sanger sequencing	^49^
	152	*Dasyprocta leporina*	Rodentia	1	*cytb*	Sanger sequencing	^49^
	152	*Dasypus novemcinctus*	Cingulata	3	*cytb*	Sanger sequencing	^49^
	152	*Mus musculus*	Rodentia	1	*cytb*	Sanger sequencing	^49^
*Ps. davisi*	1741	*Bos taurus*	Artiodactyla	5	*cytb*	Sanger sequencing	^53^
	542	*Coendou prehensilis*	Rodentia	2	*cytb*	Sanger sequencing	^50^
	542	*Dasyprocta fuliginosa*	Rodentia	1	*cytb*	Sanger sequencing	^50^
	542	*Didelphis marsupialis*	Didelphimorphia	1	*cytb*	Sanger sequencing	^50^
	90	*Gallus gallus*	Galliformes	2	*cytb*	Sanger sequencing	^51^
	491-2019	*Homo sapiens*	Primates	1	*cytb*	Sanger sequencing	^53^ ^,^ ^54^ ^,^ ^78^
	542	*Marmosops noctivagus*	Didelphimorphia	1	*cytb*	Sanger sequencing	^50^
	184-542	*Pecari tajacu*	Artiodactyla	1	*cytb*	Sanger sequencing	^50^ ^,^ ^81^
	2019	*Psophia viridis*	Gruiformes	1	*cytb*	Sanger sequencing	^54^
	184	*Sus scrofa*	Artiodactyla	1	*cytb*	Sanger sequencing	^81^
	184-2019	*Tamandua tetradactyla*	Pilosa	3	*cytb*	Sanger sequencing	^54^ ^,^ ^81^
*Ps. hirsutus*	391	*Alouatta seniculus*	Primates	1	*cytb*	Sanger sequencing	^78^
	391	*Dasypus sabanicola*	Cingulata	1	*cytb*	Sanger sequencing	^78^
	5	*Dasypus novemcinctus*	Cingulata	1	*cytb*	Sanger sequencing	^81^
	47	*Gallus gallus*	Galliformes	1	*cytb*	Sanger sequencing	^51^
	391	*Homo sapiens*	Primates	4	*cytb*	Sanger sequencing	^78^
*Ps. hirsutus hirsutus*	202	*Bos taurus*	Artiodactyla	1	*cytb*	Sanger sequencing	^53^
	86	*Bos taurus*	Artiodactyla	1	*cytb*	Sanger sequencing	^50^
*Ps. llanosmartinsi*	167	*Homo sapiens*	Primates	1	*cytb*	Sanger sequencing	^50^
*Ps. lloydi*	137	Avian (generic)	-	6	*cytb*	PCR-RFLP	^26^
	137	*Homo sapiens*	Primates	8	*cytb*	PCR-RFLP	^26^
*Ps. paraensis*	92	*Dasypus novemcinctus*	Cingulata	1	*cytb*	Sanger sequencing	^49^
*Psychodopygus* sp.	54	*Homo sapiens*	Primates	1	*cytb*	Sanger sequencing	^50^
*Sciopemyia aff. microps*	176	*Bokermannohyla martinsi*	Anura	20	COI	Sanger sequencing	^24^
	176	*Scinax fuscovarius*	Anura	1	COI	Sanger sequencing	^24^
*Sc. servulolimai*	3	*Sus scrofa*	Artiodactyla	1	*cytb*	Sanger sequencing	^50^
*Sc. sordellii*	21	*Bokermannohyla martinsi*	Anura	2	COI	Sanger sequencing	^24^
	2	*Canis lupus familiaris*	Carnivora	1	*cytb*	PCR-RFLP	^27^
	67	*Gallus gallus*	Galliformes	2	*cytb*	PCR-RFLP	^117^
	67	*Homo sapiens*	Primates	1	*cytb*	Sanger sequencing	^50^
	28	*Sus scrofa*	Artiodactyla	8	*cytb*	PCR-RFLP	^117^
*Trichophoromyia* sp.	537	*Gallus gallus*	Galliformes	11	*cytb*	Sanger sequencing	^51^
	7	*Homo sapiens*	Primates	1	*cytb*	Sanger sequencing	^50^
*Trichopygomyia* sp.	4	*Choloepus didactylus*	Pilosa	1	*cytb*	Sanger sequencing	^50^

COI: cytochrome oxidase I; *cytb*: *cytochrome b*; PCR-RFLP: polymerase chain reaction-restriction fragment length polymorphism; PNOC: prepronociceptin.

**Fig. 2:**
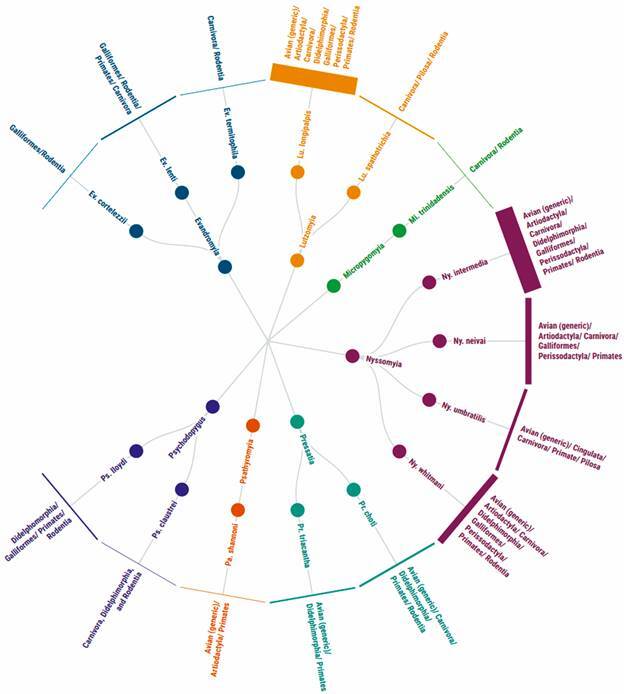
radial tree showing the relationship between the sand fly species and the vertebrate orders serving as blood source in mixed feeding reports

The precipitin test (Table IV) was featured in 8 out of 36 studies, accounting for 23% of the research, and provided information on the identification of blood sources for nine sand fly species. It generally utilised different antisera, including those for bird, armadillo, chicken, dog, goat, opossum, equine, feline, human, sheep, rodent, and pig, or employed family-specific antisera. Conversely, enzyme-linked immunosorbent assay (ELISA) was employed in 7 out of 36 included studies (19%) (Table V), and the identification of blood sources for 17 sand fly species was obtained through the test with the same spectrum of antisera used in the precipitin test. In addition to the detection of a single food source, fourteen studies (38%) employing ELISA and precipitin techniques also reported sand flies with mixed feeding, in which two or more blood sources were simultaneously detected in the same sand fly. In contrast, only two studies identified multiple feedings through PCR-RFLP targeted to the *cytb* gene.^26^ ^,^ ^27^ A total of 15 sand fly species within seven genera were found engorged with mixed feeding (Fig. 2). The genus *Nyssomyia* was the most prevalent, with four species reported with mixed feeding, followed by *Evandromyia* (3), *Psychodopygus* (2), *Lutzomyia* (2), *Pressatia* (2), *Micropygomyia* (1), and *Psathyromyia* (1). Notably, a total of at least eight vertebrate orders have been associated with *Lutzomyia longipalpis*, *Nyssomyia intermedia*, and *Nyssomyia whitmani*, demonstrating a high plasticity of these species in feeding habits.

**TABLE IV:** Blood source identification of Brazilian sand fly species by Precipitin test

Sand fly	Number/Range of specimens collected	Vertebrate host	Vertebrate host order	Number/Range of sand flies feeding on the host	Antisera used to detect the blood source	Reference
*Lu. longipalpis*	2376	Avian (generic)	NA	25	cattle, dog, horse, pig, rodent, avian and human	^(89)^
	916-2376	*Bos taurus*	Artiodactyla	4-9	cattle, horse, pig, rodent, dog, human, and avian	^(89,114)^
	916-2376	*Canis lupus familiaris*	Carnivora	2-5	cattle, horse, pig, rodent, dog, human, and avian	^(89,114)^
	916-2376	*Equus ferus caballus*	Perissodactyla	8-10	cattle, horse, pig, rodent, dog, human, and avian	^(89,114)^
	916	*Gallus gallus*	Galliformes	10	cattle, horse, pig, rodent, dog, man and chicken antisera	^(114)^
	916-2376	*Homo sapiens*	Primates	2-12	cattle, horse, pig, rodent, dog, human, and avian	^(89,114)^
	2376	Opossum (generic)	Didelphimorphia	12	cattle, dog, horse, pig, rodent, avian and human antisera	^(89)^
	916-2376	Rodent (generic)	Rodentia	6-16	cattle, horse, pig, rodent, dog, human, and avian	^(89,114)^
*Lu. spathotrichia*	34	*Bradypus* sp.	Pilosa	10	human, rodent, edentate, dog and chicken antisera	^(37)^
	34	*Canis lupus familiaris*	Carnivora	2	human, rodent, edentate, dog and chicken antisera	^(37)^
	34	*Gallus gallus*	Galliformes	6	human, rodent, edentate, dog and chicken antisera	^(37)^
	34	*Homo sapiens*	Primates	4	human, rodent, edentate, dog and chicken antisera	^(37)^
	34	*Rattus rattus*	Rodentia	5	human, rodent, edentate, dog and chicken antisera	^(37)^
*Ny. anduzei*	1	*Bradypus* sp.	Pilosa	1	human, rodent, edentate, dog and chicken antisera	^(37)^
*Ny. intermedia*	NA	Avian (generic)	NA	59-130	avian, pig, dog, cat, horse, cattle, opossum, rodent armadillo, and human	^(31)^
	370	*Bos taurus*	Artiodactyla	8	chicken, pig, dog, cat, horse, cattle, opossum, armadillo, rodent, and human	^(31,113)^
	370	*Canis lupus familairis*	Carnivora	18-26	avian, pig, dog, cat, horse, cattle, opossum, rodent armadillo, and human	^(31,113)^
	370	*Equus ferus caballus*	Perissodactyla	12-18	avian, pig, dog, cat, horse, cattle, opossum, rodent armadillo, and human	^(31,113)^
	NA	*Felis silvestris catus*	Carnivora	4	chicken, pig, dog, cat, horse, cattle, opossum, armadillo, rodent, and human	^(31)^
	370	*Homo sapiens*	Primates	22-53	avian, pig, dog, cat, horse, cattle, opossum, rodent armadillo, and human	^(31,113)^
	NA	Opossum (generic)	Didelphimorphia	38	chicken, pig, dog, cat, horse, cattle, opossum, armadillo, rodent, and human	^(31)^
	370	Rodent (generic)	Rodentia	31-80	avian, pig, dog, cat, horse, cattle, opossum, rodent armadillo, and human	^(31,113)^
*Ny. umbratilis*	161	*Canis lupus familiaris*	Carnivora	39	human, rodent, edentate, dog and chicken	^(37)^
	161	*Bradypus* sp.	Pilosa	34	human, rodent, edentate, dog and chicken	^(37)^
	161	*Gallus gallus*	Galliformes	18	human, rodent, edentate, dog and chicken	^(37)^
	161	*Homo sapiens*	Primates	31	human, rodent, edentate, dog and chicken	^(37)^
	161	*Rattus rattus*	Rodentia	59	human, rodent, edentate, dog and chicken	^(37)^
	975	-	Perissodactyla	13	family-specific	^(115)^
	975	-	Cingulata or Pilosa	674	family-specific	^(115)^
	975	-	Rodentia	185	family-specific	^(115)^
	975	-	Primates	14	family-specific	^(115)^
	975	-	Lagomorpha	15	family-specific	^(115)^
	975	-	Didelphimorphia	11	family-specific	^(115)^
	975	-	Carnivora	6	family-specific	^(115)^
	975	-	Artiodactyla	1	family-specific	^(115)^
*Ny. whitmani*	20	Avian (generic)	-	2	human, avian, cattle, horse, goat, cat, pig, rodent, opossum, armadillo, sheep, lizzard and frog	^(82,88)^
	20	*Bos taurus*	Artiodactyla	2-4	human, avian, chicken, cattle, horse, goat, cat, dog, pig, rodent, opossum, armadillo, sheep, armadillo, lizzard and frog	^(82,88)^
	20	*Equus ferus caballus*	Perissodactyla	4	human, avian, cattle, horse, goat, cat, pig, rodent, opossum, armadillo, sheep, lizzard and frog	^(82)^
	NA	*Gallus gallus*	Galliformes	14	human, chicken, cattle, dog, horse, cat, pig, rodent, opossum, armadillo, lizzard and frog	^(88)^
	20	*Homo sapiens*	Primates	2-8	human, avian, chicken, cattle, horse, goat, cat, dog, pig, rodent, opossum, armadillo, sheep, armadillo, lizzard and frog	^(82,88)^
	NA	Opossum (generic)	Didelphimorphia	7	human, chicken, cattle, dog, horse, cat, pig, rodent, opossum, armadillo, lizzard and frog	^(88)^
	20	Rodent (generic)	Rodentia	8-9	human, avian, chicken, cattle, horse, goat, cat, dog, pig, rodent, opossum, armadillo, sheep, armadillo, lizzard and frog	^(82,88)^
	NA	*Sus scrofa*	Artiodactyla	4	human, chicken, cattle, dog, horse, cat, pig, rodent, opossum, armadillo, lizzard and frog	^(88)^
*Pr. choti*	164	Avian (generic)	-	36	human, avian, cattle, horse, goat, cat, pig, rodent, opossum, armadillo, sheep, lizzard and frog	^(82)^
	164	*Bos taurus*	Artiodactyla	6	human, avian, cattle, horse, goat, cat, pig, rodent, opossum, armadillo, sheep, lizzard and frog	^(82)^
	164	*Canis lupus familiaris*	Carnivora	14	human, avian, cattle, horse, goat, cat, pig, rodent, opossum, armadillo, sheep, lizzard and frog	^(82)^
	164	*Didelphis albiventris*	Didelphimorphia	10	human, avian, cattle, horse, goat, cat, pig, rodent, opossum, armadillo, sheep, lizzard and frog	^(82)^
	164	*Equus ferus caballus*	Perissodactyla	14	human, avian, cattle, horse, goat, cat, pig, rodent, opossum, armadillo, sheep, lizzard and frog	^(82)^
	164	*Homo sapiens*	Primates	10	human, avian, cattle, horse, goat, cat, pig, rodent, opossum, armadillo, sheep, lizzard and frog	^(82)^
	164	Rodent (generic)	Rodentia	60	human, avian, cattle, horse, goat, cat, pig, rodent, opossum, armadillo, sheep, lizzard and frog	^(82)^
*Pr. triacantha*	90	Avian (generic)	-	24	human, avian, cattle, horse, goat, cat, pig, rodent, opossum, armadillo, sheep, lizzard and frog	^(82)^
	90	*Bos taurus*	Artiodactyla	4	human, avian, cattle, horse, goat, cat, pig, rodent, opossum, armadillo, sheep, lizzard and frog	^(82)^
	90	*Canis lupus familiaris*	Carnivora	2	human, avian, cattle, horse, goat, cat, pig, rodent, opossum, armadillo, sheep, lizzard and frog	^(82)^
	90	*Equus ferus caballus*	Perissodactyla	10	human, avian, cattle, horse, goat, cat, pig, rodent, opossum, armadillo, sheep, lizzard and frog	^(82)^
	90	*Homo sapiens*	Primates	4	human, avian, cattle, horse, goat, cat, pig, rodent, opossum, armadillo, sheep, lizzard and frog	^(82)^
	90	Rodent (generic)	Rodentia	28	human, avian, cattle, horse, goat, cat, pig, rodent, opossum, armadillo, sheep, lizzard and frog	^(82)^
*Pa. Shannoni* complex	951	Avian (generic)	-	NA	family-specific antisera	^(115)^
	2	*Canis lupus familiaris*	Carnivora	1	human, rodent, edentate, dog and chicken	^(37)^
	2	*Rattus rattus*	Rodentia	1	human, rodent, edentate, dog and chicken	^(37)^
	951	-	Perissodactyla	3	family-specific antisera	^(115)^
	951	-	Cingulata or Pilosa	730	family-specific antisera	^(115)^
	951	-	Rodentia	91	family-specific antisera	^(115)^
	951	-	Carnivora	26	family-specific antisera	^(115)^
	951	-	Primates	15	family-specific antisera	^(115)^
	951	-	Lagomorpha	8	family-specific antisera	^(115)^
	951	-	Didelphimorphia	3	family-specific antisera	^(115)^

NA: data not available

**TABLE V:** Blood source identification of Brazilian sand fly species by enzyme-linked immunosorbent assay (ELISA)

Sand fly	Number/Range of specimens collected	Vertebrate host	Vertebrate host order	Number/Range of sand flies feeding on the host	Antisera used to detect the blood source	Reference
*Bi. flaviscutellata*	111	Avian (generic)	-	1	bird, armadillo, opossum, dog, rodent, and human	^(124)^
*Ev. cortelezzii*	11	*Gallus gallus*	Galliformes	NA	chicken, dog, rodent, and human	^(123)^
	11	*Canis lupus familiaris*	Carnivora	NA	chicken, dog, rodent, and human	^(123)^
	11	Rodent (generic)	Rodentia	NA	chicken, dog, rodent, and human	^(123)^
*Ev. infraspinosa*	1492	Armadillo (generic)	Cingulata	1	bird, armadillo, opossum, dog, rodent, and human	^(124)^
*Ev. lenti*	14	*Homo sapiens*	Primates	5	bird, human, dog, rat, and pig	^(120)^
*Lu. almeiroi*	57	Avian (generic)	-	25	NA	^(118)^
*Lu. cruzi*	1382	-	Carnivora	1	bird, dog, skunk, primate, and rodent	^(36)^
	1382	Avian (generic)	-	17	bird, dog, skunk, primate, and rodent	^(36)^
	1382	*Canis lupus familiaris*	Carnivora	2	bird, dog, skunk, primate, and rodent	^(36)^
*Lu. longipalpis*	26-327	Avian (generic)	-	41-212	bird, chicken, dog, goat, opossum, equine, feline, human, sheep, rodent, and pig	^(35,118,120)^
	86-327	*Canis lupus familiaris*	Carnivora	29	bird, chicken, dog, goat, opossum, equine, feline, human, sheep, rodent and pig	^(35,120,123)^
	NA	*Capra hircus*	Artiodactyla	NA	bird, dog, goat, opossum, equine, feline, human, sheep, and rodent	^(35)^
	NA	*Equus ferus caballus*	Perissodactyla	NA	bird, dog, goat, opossum, equine, feline, human, sheep, and rodent	^(35)^
	NA	Feline (generic)	Carnivora	NA	bird, dog, goat, opossum, equine, feline, human, sheep, and rodent	^(35)^
	86	*Gallus gallus*	Galliformes	NA	chicken, dog, rodent, and human	^(123)^
	86-327	*Homo sapiens*	Primates	217	bird, chicken, dog, goat, opossum, equine, feline, human, sheep, pig, and rodent	^(35,120,123)^
	NA	Opossum (generic)	Didelphimorphia	NA	bird, dog, goat, opossum, equine, feline, human, sheep, and rodent	^(35)^
	NA	*Ovis aries*	Artiodactyla	NA	bird, dog, goat, opossum, equine, feline, human, sheep, and rodent	^(35)^
	86	Rodent (generic)	Rodentia	NA	bird, chicken, dog, goat, opossum, equine, feline, human, sheep, and rodent	^(35,123)^
*Ny. intermedia*	542	*Bos taurus*	Artiodactyla	3	human, chicken, dog, rat, horse, pig, and bovine	^(119)^
	542	*Equus ferus caballus*	Perissodactyla	5	human, chicken, dog, rat, horse, pig, and bovine	^(119)^
	542	*Gallus gallus*	Galliformes	17	human, chicken, dog, rat, horse, pig, and bovine	^(119)^
	542	*Homo sapiens*	Primates	24	human, chicken, dog, rat, horse, pig, and bovine	^(119)^
	542	*Sus scrofa*	Artiodactyla	210	human, chicken, dog, rat, horse, pig, and bovine	^(119)^
*Ny. neivai*	195	*Canis lupus familiaris*	Carnivora	1	human, chicken, dog, rat, horse, pig, and bovine	^(119)^
	195	*Equus ferus caballus*	Perissodactyla	4	human, chicken, dog, rat, horse, pig, and bovine	^(119)^
	195	*Gallus gallus*	Galliformes	7	human, chicken, dog, rat, horse, pig, and bovine	^(119)^
	195	*Homo sapiens*	Primates	11	human, chicken, dog, rat, horse, pig, and bovine	^(119)^
	195	*Sus scrofa*	Artiodactyla	72	human, chicken, dog, rat, horse, pig, and bovine	^(119)^
*Ny. umbratilis*	2704	Armadillo (generic)	Cingulata	1	bird, armadillo, opossum, dog, rodent, and human	^(124)^
	2704	Avian (generic)	-	8	bird, armadillo, opossum, dog, rodent, and human	^(124)^
	2704	*Homo sapiens*	Primates	1	bird, armadillo, opossum, dog, rodent, and human	^(124)^
*Ny. whitmani*	3	*Homo sapiens*	Primates	1	bird, human, dog, rat, and pig	^(120)^
*Pi. christenseni*	1	*Homo sapiens*	Primates	1	bird, human, dog, rat, and pig	^(120)^
*Pa. aragaoi*	2	*Homo sapiens*	Primates	1	bird, human, dog, rat, and pig	^(120)^
*Pa. hermanlenti*	1	Avian (generic)	-	1	bird, human, dog, rat, and pig	^(120)^
*Pa. Shannoni* complex	2	*Homo sapiens*	Primates	1	bird, human, dog, rat, and pig	^(120)^
*Ps. claustrei*	14	Armadillo (generic)	Cingulata	1	bird, armadillo, opossum, dog, rodent, and human	^(124)^
	2	*Homo sapiens*	Primates	1	bird, human, dog, rat, and pig	^(120)^
*Ps. squamiventris maripaensis*	220	Armadillo (generic)	Cingulata	4	bird, armadillo, opossum, dog, rodent, and human	^(124)^

NA: data not available.

**Fig. 3:**
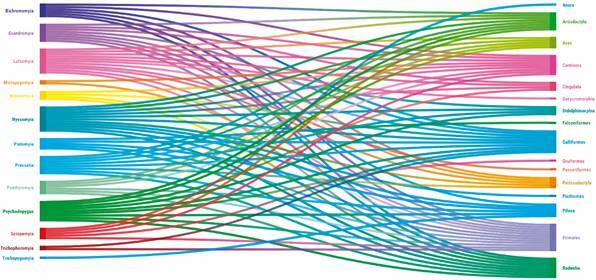
interaction matrix (Sankey diagram) between sand fly genera and vertebrates identified as the food source.

A complex interaction matrix was observed between the sand fly genera and the orders of vertebrates identified in the blood meal (Fig. 3). For all sand fly genera, except *Trichopygomyia*, more than one vertebrate order has been associated. The *Nyssomyia* genus accounted for 94 single blood meal identifications, with *Ny. whitmani*, *Ny. intermedia*, and *Nyssomyia umbratilis* being the most representative species. The *Lutzomyia* genus followed, with a high number of blood meal identifications attributed mainly to *Lu. longipalpis*. Notably, *Psychodopygus* also contributed significantly with 42 single blood meal identifications for at least ten species.

**Fig. 4:**
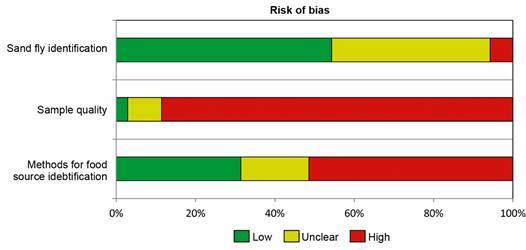
potential risk of bias identified in the included studies

*Risk of bias* - Considering the three domains evaluated, a high risk of bias was mainly observed in the domain related to the selection and preservation of engorged females (Fig. 4). This is primarily associated with the lack of information regarding the female’s level of engorgement (partial or full) and the stage of the digestive process (recent or late). The risk of bias observed regarding the methodology used to identify the food source is mainly associated with the absence of controls (*e.g.*, male sand flies as endogenous controls) and the lack of a predefined cut-off point for ELISA and precipitin techniques, or minimal similarity criteria attributed to the amplicon compared to the GenBank database. A low risk of bias was found in the domain of identifying sand flies, as few studies did not report using a specific classification key.

## DISCUSSION

Although leishmaniasis has been known to occur in Brazil since the early 1900s,^28^ it was predominantly in the last two decades that blood source identification of sand flies emerged as a subject of study in the country. Understanding the feeding habits of sand flies is of utmost relevance, as it provides indirect data regarding the presence of vertebrates that may act as potential reservoirs/hosts of *Leishmania*.^29^ The intricate relationship between sand flies and vertebrates can also be used as evidence to suspect the role of sand flies as vectors.^30^ This is also applicable to sand flies that feed exclusively on animals not hosting *Leishmania*, as knowledge of their ecological habits is of interest. This systematic review gathered data on at least 54 sand fly species distributed across 17 Brazilian states (65%), underscoring the need for continuous study, particularly in the Southern region of the country, where only one study has been conducted.^31^

Light traps were the most utilised method in studies focusing on identifying female blood sources (91.6%) compared to other collection types such as manual or Shannon traps. This fact caught our attention since fed females are not typically attracted by light and are often observed resting after blood feeding.^32^ This may account for the discrepancies between the total number of females collected and the total number of engorged females collected in the included studies (Table II). Regarding Shannon traps, in addition to light, the collector may also act as an attractant, leading to the capture of species nearby or attempting to feed on humans, mainly due to body temperature and the release of carbon dioxide through breathing. However, this attractiveness may not necessarily represent efficient feeding behaviour. Overall, it is evident that the sand fly collection method used to assess the blood meal source of females warrants further discussion.

Various techniques for identifying blood sources in sand flies have been employed, with molecular methods predominating alongside precipitin and ELISA techniques. The detection of mixed feedings underscores the need for careful consideration of the techniques used, particularly in assessing specificity and cross-reactivity. Overall, blood source identification is especially pertinent as a complementary tool in entomological studies aiming to identify and characterise potential vectors in endemic areas. In this regard, based on the results obtained here, we aim to explore and discuss two crucial points: methods for identifying blood sources and ecological inferences derived from blood sources.

*Blood source identification in sand flies: methodological considerations* - Similar to other dipterans, sand flies generally exhibit gonotrophic concordance, wherein each blood meal is followed by oviposition.^32^ The duration of this process may vary among species, but it typically occurs within 5-7 days.^33^ Complete blood meal digestion usually takes place within 72-96 hours after blood feeding, and the degree of digestion can be a crucial factor in identifying the food source. Blood digestion is commonly categorised into three levels: 1) fresh blood (bright red content in the midgut with intact erythrocytes visible under the microscope), 2) partially digested blood (dark red), and 3) extensively digested blood (brown).^34^ Interestingly, only four studies within the selected articles have characterised the level of engorgement or the stage of the digestive process.^35^ ^,^ ^36^ ^,^ ^37^ ^,^ ^38^ This lack of information is particularly noteworthy because, in general, the studies reported a higher number of females assumed to be engorged compared to females in which the food source was identified. This discrepancy may be attributed to the amount of blood ingested or the level of blood digestion, which, if extensively digested, could lead to the failure to identify the food source.^39^ However, studies are necessary to ascertain the optimum range for detecting the blood source using different methodologies, such as ELISA and the precipitin test, even though estimating this variable for wild-caught females is challenging.

One of the primary challenges posed by sand flies as a molecular study model is the size of their tissue/body, which hampers the isolation of sufficient DNA for amplifying specific molecular targets. The amount of blood ingested by a female in a single feeding is not well-established; however, it is suggested to be equivalent to the insect’s own weight, which can vary from 0.1 to 0.6 mg.^40^ Typically, full engorgement occurs in a single feeding, but occasionally a partly engorged sand fly may relocate to another site and continue feeding. Therefore, when considering sand flies that have fed on various vertebrates, the amount of blood ingested from each source can vary, influencing DNA recovery and consequently limiting the ability to identify mixed feedings. Conversely, the reactivity of blood containing multiple IgG from distinct hosts to antisera used in both the precipitin and ELISA tests appears to be more sensitive, allowing for the identification of mixed feedings.

Concerning ELISA and precipitin techniques, all studies utilised antisera from various vertebrates (*e.g.*, cattle, horse, pig, rodent, human, chicken, etc.) to detect specific antibodies for identifying the blood source. However, a high risk of bias was observed in most studies due to the absence of specificity evaluations, potentially impacting cross-reactivity and consequently allowing the detection of mixed feedings. Notably, no instances of mixed feeding were observed when Sanger sequencing was employed. A potential explanation for this phenomenon could be the challenge of identifying polymorphic sites within the amplicon or the quantity of DNA obtained after the DNA extraction step. In either scenario, cloning the PCR product might be a useful strategy to enhance the detection of multiple blood feedings.

The volume of blood available for DNA extractions serves as a clear indicator of DNA yield.^41^ Long-term storage conditions of sand fly tissue/blood, a crucial factor in molecular studies,^42^ can impact DNA integrity.^43^ Appropriate storage conditions depend on various factors, including the intended analyses and the duration of specimen storage. Generally, blood samples stored at 4ºC for a short period still yield DNA of acceptable quality if the correct blood collection tubes are used,^44^ ^,^ ^45^ although this practice is not standard in entomological studies. The preferred temperature for long-term DNA isolation from whole blood samples is -80ºC.^43^ While whole blood samples can also be frozen at -20°C for long-term storage,^43^ ^,^ ^46^ ^,^ ^47^ some studies have reported lower DNA yields with this approach.^44^ ^,^ ^47^ Regarding the blood content within sand flies, there is no evidence suggesting the optimal storage method, which makes the risk of bias related to this aspect unclear. However, it appears that temperatures higher than -80ºC may compromise blood integrity during long-term storage, potentially affecting the determination of the food source using both molecular methods and specific antibodies.

The storage method appears to be a concern in blood-feeding studies; however, seven articles (19%) did not specify the preservation method. Twelve studies (33%) reported that sand flies were stored dry after dissection at various temperatures, such as -7ºC, -15ºC, and -20ºC. In these cases, long-term storage may affect blood quality and, consequently, DNA recovery.^43^ Ethanol was employed to preserve sand fly samples, with varying concentrations, including 70%,^24^ ^,^ ^25^ ^,^ ^48^ ^,^ ^49^ 80%,^50^ 90%,^51^ and 96%.^52^ ^,^ ^53^ ^,^ ^54^ The stability of ethanol in stored blood is of immense entomological interest, as there is often a need for the analysis of such blood samples months after collection. Although some authors stored sand flies preserved in ethanol at different temperatures (-10ºC and -20ºC), the quality of blood in alcohol samples appears not to be affected at these temperatures for at least six months.^55^ Dimethyl sulfoxide (DMSO) at 6% was used to preserve engorged females in two studies^26^ ^,^ ^56^ and seems to be an efficient and cost-effective preservative method, as it protects cells from intracellular ice formation-induced damage, acting as a cryoprotective agent.^57^

In summary, these findings emphasise the need for standardised methodologies and meticulous consideration of storage conditions in future entomological studies to enhance the accuracy and reliability of blood source identification in sand flies.

*Ecological inferences based on blood sources* - Over time, numerous studies have explored the feeding tendencies of sand flies, often relying on attractiveness to vertebrates as a proxy for feeding preferences. However, such studies are limited as they do not always identify the actual blood sources, making the data fragile due to the potential for misinterpretation. For instance, while it has been suggested that species within the *Martinsmyia* genus are attracted to rodents based on bait studies, the blood sources of these species remain unidentified. Similarly, species within the *Micropygomyia* and *Sciopemyia* genera, traditionally believed to be exclusively attracted to cold-blooded animals, have been found feeding on warm-blooded animals, indicating variable feeding habits. The association between sand fly feeding habits and the detection of *Leishmania* parasites further complicates our understanding of their role in disease transmission. Although human blood has been detected in sand fly species from the *Micropygomyia* and *Sciopemyia* genera, the observed anthropophilic behaviour does not necessarily confirm their role as vectors, emphasising the necessity for more comprehensive studies. Furthermore, the possibility of cross-contamination of samples with human DNA during sand fly processing should be taken into consideration. In the risk of bias assessment, studies reporting descriptions regarding the use of sterile materials during sand fly dissection were considered as having a low risk of bias in the Sample Quality domain.

Human blood was detected in at least 35 sand fly species, making it the most identified vertebrate, followed by *Gallus gallus* blood, detected in 25 species. Chickens are refractory to *Leishmania* infection; however, their presence in peridomestic areas is often suggested as a risk factor of visceral leishmaniasis (VL). Belo et al.^58^ conducted a meta-analysis of factors associated with VL and found conflicting data. On one hand, the presence of a chicken coop may attract sand flies,^14^ thereby increasing the chances of dogs (the main *L. infantum* reservoir) being bitten by them. On the other hand, if the vector feeds on chickens, it could reduce the proportion of effective bites on dogs. Given these contradictory results, further studies are needed to clarify this relationship. Moreover, the fact that sand flies have fed on various bird orders may indicate their ecological habits, such as utilising tree canopies for opportunistic blood feeding.

Additionally, while certain vertebrates like Artiodactyls and Equines may not be considered primary hosts of *Leishmania* parasites, their presence can influence sand fly density and human-sand fly exposure, thereby impacting disease transmission dynamics.^59^ Noteworthy, *Leishmania* (*Mundinia*) *orientalis* has been reported in bovines from Switzerland^60^ and descriptions of a horse infected by *Leishmania braziliensis* in South America was in the first half of the last century.^61^ Since then, several studies have suggested the presence of this parasite in horses and donkeys from Brazil.^62^ ^,^ ^63^ ^,^ ^64^ *Leishmania infantum* infection has also been reported in equines, causing skin lesions and locomotor problems,^65^ but it is also associated with asymptomatic disease.^66^ ^,^ ^67^ Representative species of the subgenus *Mundinia* ^68^ have been identified in horses from Florida (USA) and Rio de Janeiro (Brazil),^69^ ^,^ ^70^ indicating that this vertebrate should sporadically represent a relevant role as a host of these parasites. However, studies assessing the competence of these animals as reservoirs should be conducted.

Most of the blood feeding reports on Carnivora were represented by feedings on *Canis lupus familiaris* (domestic dog), widely known to be the primary reservoir of *L. infantum* in urban areas of Brazil,^71^ and the remaining were associated with *Felis silvestris catus* (domestic cat), in which *L. infantum* infections have also been sporadically reported.^72^ Other sylvatic Carnivora, like the crab-eating fox (*Cerdocyon thous*) and the bush dog (*Speothos venaticus*), have also been suggested as reservoirs of *L. infantum*.^73^ Several sand fly species, such as *Bichromomyia flaviscutellata*, *Lutzomyia cruzi*, *Lu. longipalpis*, *Migonemyia migonei*, *Ny. intermedia*, *Nyssomyia neivai*, *Ny. umbratilis*, and *Ny. whitmani*, have been found feeding on dogs, and there is ongoing debate regarding their role as vectors of *Leishmania*.^74^ ^,^ ^75^ However, the presence of other species feeding on dogs, such as *Evandromyia cortelezzii*, *Evandromyia evandroi*, *Evandromyia termitophila*, and *Pressatia choti*, opens perspectives on the capacity and competence of vectorial studies to ascertain their involvement in the epidemiological cycle, particularly of *L. infantum*.

The nine-banded armadillo (*Dasypus novemcinctus*) is considered a potential reservoir of *Leishmania naiffi* in Brazil.^76^ ^,^ ^77^ At least five sand fly species have been found feeding on this vertebrate (*Evandromyia* sp.; *Psychodopygus ayrozai*, Chagasi series of *Psychodopygus* genus, *Psychodopygus paraensis*, and *Psychodopygus carrerai carrerai*) in studies conducted in Roraima and Acre, both in the Northern region of Brazil,^49^ ^,^ ^50^ consistent with the geographical distribution of *L. naiffi*. Of these sand fly species, only *Ps. ayrozai* has been suggested as a putative vector of this parasite.^74^ Other Cingulata have also been found as blood sources for sand flies, such as *Dasypus sabanicola*,^78^ and the six-banded armadillo *Euphractus sexcinctus*;^79^ however, there is a lack of information regarding their role as hosts/reservoirs of *Leishmania*.

The participation of other infected mammals, rather than dogs, in the transmission cycle of *L. infantum* in urban areas, has already been proposed for opossums (Didelphimorphia).^80^ A total of eight sand fly species, within five genera (*Lutzomyia*, *Nyssomyia*, *Pressatia*, *Psathyromyia*, and *Psychodopygus*), have been found feeding on at least four species of opossums: *Philander canus*, *Didelphis albiventris*, *Didelphis marsupialis*, and *Marmosops noctivagus*.^50^ ^,^ ^81^ ^,^ ^82^ Of these, only *D. albiventris* and *D. marsupialis* are considered potential reservoirs of *L. infantum* and *Leishmania guyanensis*, respectively.^83^ ^,^ ^84^ ^,^ ^85^ However, other species of the genera *Philander* and *Marmosops* have also been suspected of sustaining *Leishmania amazonensis* and *L. guyanensis* infection, respectively.^86^ ^,^ ^87^ In general, opossums are synanthropic and are frequently found in peridomiciliary areas, where they may serve as a source of infection to vectors, such as *Lu. longipalpis*, *Ny. intermedia*, and *Ny. whitmani*, which have been found feeding on opossums.^27^ ^,^ ^31^ ^,^ ^35^ ^,^ ^81^ ^,^ ^88^ ^,^ ^89^

The order Pilosa, a clade of xenarthran placental mammals, includes anteaters and sloths.^90^ Together with marsupials, these ancient *Leishmania* hosts are also native American fauna and possess a peculiar blood vessel structure that allows for an extremely low metabolic rate, conserving energy.^91^ Regarding anteaters, only *Tamandua tetradactyla*, a putative host of *L. amazonensis*, *L. guyanensis*, and *L. infantum*,^92^ ^,^ ^93^ ^,^ ^94^ has been reported as a blood source for *Evandromyia*, *Nyssomyia*, *Psathyromyia*, and *Psychodopygus*.^48^ ^,^ ^50^ ^,^ ^52^ ^,^ ^53^ ^,^ ^54^ ^,^ ^81^ The two genera of sloths (*Bradypus* and *Choloepus*) have representatives considered putative reservoirs of *Leishmania* in Brazil. *Bradypus tridactylus* has been associated with *L. shawi* infections, and *Choloepus didactylus* has been associated with *L. guyanensis* in the Northern region.^95^ Moreover, *C. hoffmanni* has been associated with *Leishmania colombiensis* and *Leishmania equatoriensis* (syn = *Endotrypanum colombiensis* and *Endotrypanum equatoriensis*, respectively)^68^ in South America.^96^ ^,^ ^97^ Six sand fly species (*Lutzomyia spathotrichia*, *Nyssomyia anduzei*, *Ny. umbratilis*, *Ny. antunesi*, *Pintomyia fiocruzi*, and *Trichopygomyia* sp.) have been found feeding on sloths, and among these species, *Ny. anduzei* and *Ny. umbratilis* have been reported as naturally infected with *Endotrypanum* ^97^ ^,^ ^98^ and may be considered putative vectors of these parasites.

Among primates, sand flies feeding on humans suggest anthropophilic tendencies, but their role as vectors remains debated. Notably, *Micropygomyia trinidadensis* and *Sciopemyia sordellii*, frequently associated with cold-blooded animals, have been found feeding on humans.^27^ ^,^ ^50^ Molecular analysis has shown the presence of *L. amazonensis*, *L. infantum*, and *L. braziliensis* DNA in *Mi. trinidadensis* ^99^ and *L. braziliensis*, *L. infantum*, and *L. naiffi* in *Sc. sordellii*.^100^ ^,^ ^101^ Until today, the role of members of *Micropygomyia* and *Scyopemia* as vectors of *Leishmania* is debated. The presence of blood from *Alouatta seniculus* (Venezuelan red howler) and *Plecturocebus bernhardi* (zog-zog monkey) was also detected in *Psychodopygus hirsutus*, *Ny. antunesi* complex, and *Ny. umbratilis*; however, these vertebrates are not yet considered hosts/reservoirs of *Leishmania*.

Nine species of rodents belonging to six genera have been detected as blood sources for sand flies. Blood from three species of agouti (*Dasyprocta azarae*, *Dasyprocta leporina*, and *Dasyprocta fuliginosa*) has been detected in *Lu. cruzi*, *Ny. antunesi*, *Psychodopygus davisi*, and the Chagasi series of the *Psychodopygus* genus, with only *D. azarae* considered a putative host of *L. infantum*.^73^ The lowland paca (*Cuniculus paca*) is considered a putative host of *Leishmania lainsoni*,^102^ and its blood was detected within *Ny. antunesi*,^48^ *Pintomyia serrana* and Chagasi series of *Psychodopygus* genus.^49^ ^,^ ^50^ However, although *Ny. antunesi* has been found associated with several *Leishmania* (*Viannia*) parasites,^50^ ^,^ ^54^ ^,^ ^103^ ^,^ ^104^ the presence of *L. lainsoni* has never been detected in these sand flies, only in *Evandromyia evandroi, Lu. longipalpis*, *Ny. whitmani*, *Trichophoromyia brachipyga*, and *Trichophoromyia ubiquitalis*,^100^ ^,^ ^105^ ^,^ ^106^ ^,^ ^107^ ^,^ ^108^ highlighting the necessity of further studies to understand the transmission dynamics of this parasite. *Coendu prehensilis* (Brazilian porcupine), whose blood was detected in *Pi. serrana* and *Ps. davisi*,^50^ is considered a putative host of *L. infantum* in Bolivia.^109^ *Leishmania hertigi* [syn. *Porcisia hertigi* ^68^] has been described from this vertebrate in Panama;^110^ however, in Brazil, there is no data regarding its putative role in the transmission cycle of trypanosomatids. *Rattus rattus* (black rat) blood was found in *Lu. spathotrichia*, *Ny. umbratilis*, *Pa.* Shannoni complex, and *Mg. migonei*. This rodent is known to be a reservoir of *L. braziliensis*, and these sand flies should be investigated as putative vectors. Among these sand flies, at least *Mg. migonei* is considered a permissive vector, able to sustain late-stage infections of *L. braziliensis*.^111^ Blood from the house mouse (*Mus musculus*) was found within females of Chagasi series of *Psychodopygus* genus,^49^ and this vertebrate is considered a putative host of *L. braziliensis* in peridomestic areas.^112^ In sylvatic areas, representatives of *Proechimys* seem to be hosts of *L. amazonensis* and *L. guyanensis,* ^85^ ^,^ ^86^ and blood from *Proechimys gardneri* was found in *Bi. flaviscutellata*,^52^ the primary vector of *L. amazonensis* in Brazil,^74^ evidencing a close relationship between hosts and vectors.

In conclusion, ecological inferences drawn from blood sources, in association with the presence of *Leishmania* in putative hosts, shed light on the complex dynamics of sand fly ecology. This underscores the importance of comprehensive studies to elucidate the role of sand flies in disease transmission cycles. By further investigating these ecological relationships, researchers can contribute to the development of more effective vector control strategies, ultimately helping to mitigate the burden of leishmaniasis in endemic regions.

Concluding remarks

In summary, this study underscores the critical importance of blood source identification in sand fly research to elucidate the intricate dynamics of vector-host-parasite interactions. The implementation of standardised methodologies, coupled with meticulous attention to storage conditions and the level of blood digestion in females, is paramount for advancing our comprehension of sand fly feeding ecology and its implications for *Leishmania* transmission dynamics. By confronting these methodological challenges head-on, future investigations may make significant strides in unravelling the nuanced roles of sand fly species as vectors, and by extension, their potential hosts/reservoirs, within the complex epidemiological network of *Leishmania* in Brazil.
