# Supplementary material for: You are what you eat: a systematic review exploring the interaction between Brazilian sand flies and their vertebrate food sources
Source: Mem Inst Oswaldo Cruz. 2024 Aug 30;119:e240055. doi: 10.1590/0074-02760240055 (PMC11368077; doi:10.1590/0074-02760240055)
Supplement: Supplementary file 1 [file 1678-8060-mioc-119-e240055-s.pdf]

TABLE

Search strategies used in each database

| Database | Search strategy | File |
|---|---|---|
| MEDLINE (by Pubmed) | Population:<br>#1 "insect vectors"[MeSH Terms] OR "insect vectors"[Title/Abstract] OR "insect vector"[Title/Abstract] OR "vector insect"[Title/Abstract] OR "vectors insect"[Title/Abstract] OR "Psychodidae"[MeSH Terms] OR "Psychodidae"[Title/Abstract] OR "Sandflies"[Title/Abstract] OR "sand flies"[Title/Abstract] OR "Sandfly"[Title/Abstract] OR "sand fly"[Title/Abstract] OR "phlebotomines"[Title/Abstract] OR "phlebotomine"[Title/Abstract]<br>Intervention:<br>#2 "feeding behavior"[MeSH Terms] OR "feeding behavior"[Title/Abstract] OR "behavior feeding"[Title/Abstract] OR "feeding behaviors"[Title/Abstract] OR "feeding patterns"[Title/Abstract] OR "feeding pattern"[Title/Abstract] OR "pattern feeding"[Title/Abstract] OR "food preferences"[MeSH Terms] OR "food preferences"[Title/Abstract] OR "food preference"[Title/Abstract] OR "preference food"[Title/Abstract] OR "preferences food"[Title/Abstract] OR "blood/parasitology"[MeSH Terms] OR "Blood"[MeSH Terms] OR "Blood"[Title/Abstract] OR "blood-meal"[Title/Abstract] OR "blood-meal"[Title/Abstract] OR "food source"[Title/Abstract]<br>Location:<br>#3 "Brazil"[MeSH Terms] OR "Brazil"[Title/Abstract] OR "Brazilian"[Title/Abstract] OR "American"[Title/Abstract]<br><br>#4 #1 AND #2 AND #3 | 617 |
| Web of Science | Population:<br>#1 ("insect vectors" OR "insect vector" OR "vector insect" OR "vectors insect" OR "Psychodidae" OR "Sandflies" OR "sand flies" OR "Sandfly" OR "sand fly" OR "phlebotomines" OR "phlebotomine"))<br>Intervention:<br>#2 ("feeding behavior" OR "behavior feeding" OR "feeding behaviors" OR "feeding patterns" OR "feeding pattern" OR "pattern feeding" OR "food preferences" OR "food preference" OR "preference food" OR "preferences food" OR "blood/parasitology" OR "Blood" OR "blood-meal" OR "food source"))<br>Location:<br>#3 ("Brazil" OR "Brazilian" OR "American")<br>#4 #1 AND #2 AND #3 | 543 |
| Virtual Health Library (VHL) | Population:<br>#1 (mh:(Insetos Vetores)) OR (mh:(Insectos Vectores)) OR (mh:(Insect Vectors)) OR (Insetos Vetores) OR (Insectos Vectores) OR (Insect Vectors) OR (mh:(Psychodidae)) OR (Psychodidae) OR (Phlebotominae) OR (Psychodinae) OR (Sandflies) OR (sand flies) OR (Sandfly) OR (Sand fly) OR (phlebotomines) OR (phlebotomine)<br><br>Intervention:<br>#2 (mh:(Comportamento Alimentar)) OR (mh:(Conducta Alimentaria)) OR (mh:(Feeding Behavior)) OR (Comportamento Alimentar) OR (Conducta Alimentaria) OR (Feeding Behavior) OR (Comportamento Relacionado à Alimentação) OR (Padrões Alimentares) OR (Tradições Alimentares)<br><br>Location:<br>#3 (mh:(Brasil)) OR (mh:(Brazil)) OR (Brasil) OR (Brazil)<br><br>Bases available after withdrawing MEDLINE in the filter:<br>#4 (db:("LILACS" OR "SES-SP" OR "colecionaSUS" OR "VETINDEX"))<br><br>#5 #1 AND #2 AND #3 AND #4 | 54 |
| Total | | 1,214 |

CC BY
